# Where Artificial Intelligence Can Take Us in the Management and Understanding of Cancerization Fields

**DOI:** 10.3390/cancers15215264

**Published:** 2023-11-02

**Authors:** Carmen Orte Cano, Mariano Suppa, Véronique del Marmol

**Affiliations:** 1 Department of Dermatology, Hôpital Erasme, HUB, Université Libre de Bruxelles, 808 Route de Lennik, 1070 Brussels, Belgium; 2 Department of Dermato-Oncology, Institut Jules Bordet, HUB, Université Libre de Bruxelles, 1070 Brussels, Belgium; 3 Groupe d’Imagerie Cutanée Non Invasive (GICNI), Société Française de Dermatologie (SFD), 75008 Paris, France

**Keywords:** cancerization field, actinic keratosis, squamous cell carcinoma, non-invasive imaging, line-field confocal optical coherence tomography, LC-OCT, artificial intelligence

## Abstract

**Simple Summary:**

Squamous cell carcinoma (SCC) is most often preceded by a lesion called actinic keratosis (AK) and is largely due to ultra-violet radiation exposure. Usually, these cancers appear in areas that have been ‘damaged’ by the sun, otherwise known as ‘cancerization fields’, where sub-clinical (invisible to the naked eye), precursor (AK) and cancerous (SCC) lesions coexist. For clinicians, differentiating between the three is not always easy. To facilitate these diagnoses, we dispose now of non-invasive skin imaging techniques that are comparable to a virtual biopsy. The very recent introduction of artificial intelligence could enable us to broaden the application of these technologies when applied to cancerization fields, predicting the risk of malignant transformation of precancerous lesions, guiding treatments and better understanding the mechanisms behind.

**Abstract:**

Squamous cell carcinoma and its precursor lesion actinic keratosis are often found together in areas of skin chronically exposed to sun, otherwise called cancerisation fields. The clinical assessment of cancerisation fields and the correct diagnosis of lesions within these fields is usually challenging for dermatologists. The recent adoption of skin cancer diagnostic imaging techniques, particularly LC-OCT, helps clinicians in guiding treatment decisions of cancerization fields in a non-invasive way. The combination of artificial intelligence and non-invasive skin imaging opens up many possibilities as AI can perform tasks impossible for humans in a reasonable amount of time. In this text we review past examples of the application of AI to dermatological images for actinic keratosis/squamous cell carcinoma diagnosis, and we discuss about the prospects of the application of AI for the characterization and management of cancerization fields.

## 1. Introduction

**Figure 1:**
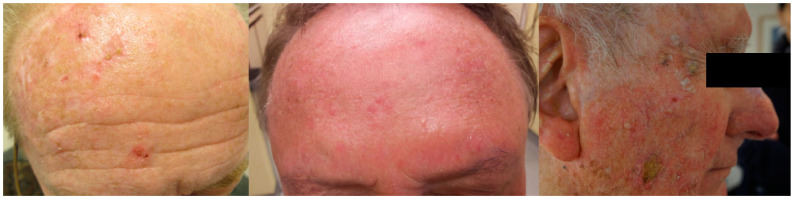
Illustration of cancerization fields where subclinical damage, actinic keratosis and early squamous cell carcinomas coexist.

Squamous cell carcinoma (SCC) of the skin is often preceded, or at least accompanied in close vicinity, by the precursor lesion actinic keratosis (AK). These cancers result from the malignant transformation of squamous cells within the epidermis, usually as a result of exposure to oncogenic ultraviolet radiation (UVR). In daily practice, clinicians are often confronted with not only solitary lesions but also areas of sun-damaged skin where clinical (AK and SCC) and subclinical damage coexist, otherwise known as cancerization fields (Figure 1).

To the naked, even expert eye, cancerization fields are difficult to characterize.

From our experience, the adoption of new skin cancer diagnostic imaging techniques helps clinicians in distinguishing precancerous and cancerous lesions in these fields. These techniques depend, however, on trained human intervention that can sometimes be subjective. The ability of computer-aided diagnosis and, more particularly, deep and machine learning artificial intelligence (AI) to help guide clinicians when using these new technologies is now being studied. We will review past attempts and future applications of new AI for cancerization fields.

## 2. Cancerization Field: Definition, Controversies and Challenges

Clinically, a cancerization field of the skin has been defined as a “multifocal clinical atypia characterized by AKs or SCCs in situ with or without invasive disease, occurring in a field exposed to chronic UVR” [1]. At the cellular level and from an evolutionary perspective, Curtius et al. defined a cancerization field as a collection of cells, with many genetically distinct clones, that have gained some but not all of the phenotypic alterations required for malignancy. In the field, mutant and cancerized lineages coexist, and from these, the cell presenting the fittest phenotype and the adequate microenvironment would dominate and eventually lead to cancer [2].

Thus, while the majority of AKs in these fields remain benign or even regress spontaneously, a subset of them can progress to invasive SCC, which has metastatic potential. Still, the risk of progression of an individual AK to an invasive SCC is difficult to determine and reports are variable across studies, with progression rates ranging from 0% to 0.53% per lesion-year [3,4]. Mostly because of this risk, current guidelines support the treatment of all AKs. It is, however, controversial, as others point out the associated morbidity and costs of these interventions, especially when taking into account the low rate of progression and non-negligible rate of spontaneous regression. We believe it is relevant for this discussion to focus on cancerization fields separately, as they present a particular diagnostic challenge and they are associated with greater treatment morbidity and costs compared to isolated lesions.

In current clinical practice, most dermatologists distinguish between AK and SCC within a field based on clinical and dermoscopic criteria. There are clinical factors that are commonly considered when assessing risk of progression, such as (i) the presence of multiple AKs; (ii) their persistence or recurrence after treatment; (iii) the location of AKs on the body, particularly those occurring on the upper extremities, lips and ears; and (iv) patients’ characteristics such as history of immunosuppression or of other non-melanoma skin cancers [5,6]. Other characteristics not necessarily related to the risk of progression to SCC are considered when assessing the need for treatment, such as risky or difficult-to-treat localizations (mostly the head), the size or thickness of a field, and treatment based upon the patient’s demand. In regard to the dermoscopic criteria, which remain our first-line diagnostic tool, white structureless areas, dotted/glomerular vessels and hairpin vessels have been shown to be the best predictors of early SCC arising from AK [7].

It is our view that the decision to treat is usually made based on the dermatologist’s experience and inclinations and that these are sometimes subjective. Indeed, a study showed that one in twenty-five lesions identified as AK within a field by a trained dermatologist were actually occult SCCs [8].

To overcome this subjectivity and to reduce the associated morbidity and costs, the application of AI to non-invasively acquired images could help in the future by guiding appropriate management strategies and by providing an accurate risk assessment.

## 3. Computer-Aided Diagnosis: Past and Future

The first attempts in the application of computer-aided diagnostic tools for the discrimination of AKs came from their application to clinical photographs [9, 10, 11, 12], where computers distinguished AKs from normal skin based on color and texture. However, aside from their potential application in teledermatology screening, these tools are not suitable to fully study and efficiently guide the treatment of cancerization fields and have been abandoned. It is known now that clinical scoring of AKs does not correlate with histological findings nor with the risk of progression to SCC [13]. What is more, clinical pictures are difficult to homogenize and can be affected by variations in the lighting, positioning and resolution.

More interestingly, the introduction of non-invasive cellular-resolution imaging technologies have paved the way for a new application of AI: the assessment of histological diagnostic and progression markers. Histological markers for the risk of progression from AKs to SCCs exist. Past publications have pointed out that AKs with atypical keratinocytes restricted to the lower third of the epidermis are the ones most at risk of transformation into SCC [14,15], debunking the hypothesis of a sequential progression (classic pathway) in which AKs with basal atypia would progress into full-thickness atypia and then into invasive SCC [16]. This has been recently supported by Falkenberg et al. who found that AKs from organ transplant recipients, known to be at high risk of developing invasive SCC, presented significantly more acantholysis and a more marked basal proliferation than non-immunosuppressed patients [16].

The new skin imaging techniques, mainly reflectance confocal microscopy (RCM), high-definition optical coherence tomography (HD-OCT) and line-field confocal optical coherence tomography (LC-OCT), have been used to assess the skin at the cellular level and in vivo, and have demonstrated good results and correlation with conventional histology, enhancing diagnostic accuracy and facilitating early intervention without the need of a biopsy. RCM provides the best resolution (1 µm) but only allows the visualization of the skin on the horizontal plane, making the evaluation of the dermal–epidermal junction difficult. The penetration depth is also the shallowest of all three techniques (up to 250 µm), strongly limiting the assessment of keratinizing carcinomas, which are usually thick. HD-OCT and LC-OCT provide more intuitive histology-like images. The degree of atypia and its localization on the epidermis can be evaluated fully on the same image. The key difference between HD-OCT and LC-OCT lies in the tradeoff between resolution and penetration depth. The first can reach a depth of 2 mm but with a poor cellular resolution, whilst the second only reaches 500 µm depth but with 1.3 µm of axial resolution and 1.1 µm of lateral resolution, almost equivalent to those of RCM.

Their current use in dermatology depends mainly on a center’s preferences and expertise, and they are used for diagnoses in daily practice in a non-automated way [17, 18, 19, 20]. For each technique, a set of diagnostic criteria has been defined (as for dermoscopy). Clinicians evaluate images and make a diagnosis based on criteria sets that are more or less standardized or adopted, but that remain qualitative and subject to interobserver variability.

On the other hand, the evaluation of atypia, or AK grading, using skin imaging methods has not been standardized and is not yet used routinely; some studies on the subject do exist though. Pellacani et al. ranked keratinocyte atypia in RCM images and in their corresponding histopathology and found a good concordance [21]. They failed, however, to objectively define atypia and to allow the reproducibility of their scoring. In another pilot study including 50 lesions, Ruini et al. used LC-OCT to evaluate the growth patterns of basal keratinocytes based on the PRO classification, which takes into account the previously described histological marker of progression [15,22]. They found a good overall classification agreement between LC-OCT and histological examinations (agreed in 75% of the observations). Even better results were found when clustering PRO-I and PRO-II/III, the highest progression risk category (85.4% concordance). Interobserver agreement was 90%, supporting the reproducibility of the technique among expert observers [23].

**Figure 2:**
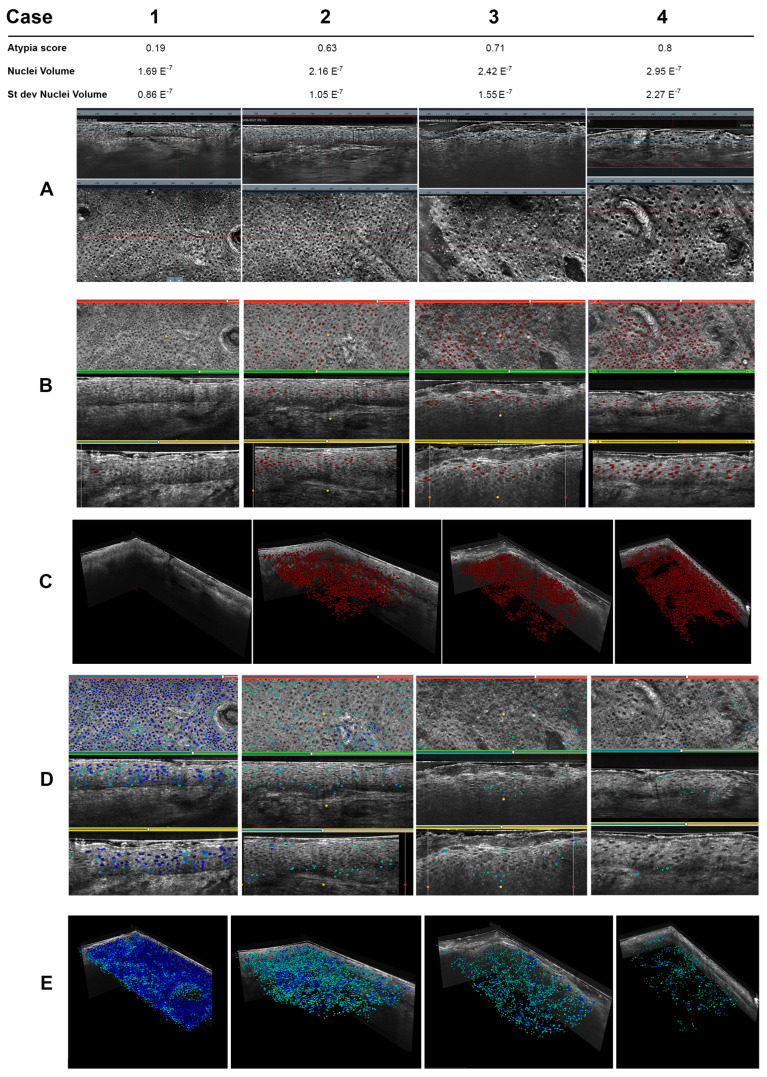
Example of a comparative review of AI atypia scores on AKs imaged with LC-OCT. Columns (1, 2, 3, 4) each show a case of AK. AI segmented nuclei into 3D images of each AK and provided an overall score of atypia considering cell metrics (nuclei volume). The spatial localization can be assessed. (**A**) LC-OCT vertical (up) and horizontal (down) views. From left to right, increase in atypia and epidermis disorganization matching AI metrics and atypia score. (**B**) Number of nuclei with the highest atypia score (red dots) and visualization of their distribution within the epidermis. (**C**) Isolation of cells with the highest atypia score. (**D**) Number of nuclei with the lowest atypia score (blue and green dots) and visualization of their distribution within the epidermis. (**E**) Isolation of cells with the highest atypia score.

Computer-aided diagnostic systems have been applied to images acquired with non-invasive techniques as they have been for clinical pictures, showing interesting results. In 2016, Boone et al. applied a simple algorithm on HD-OCT images to discriminate AK subtypes from normal skin and SCC based on optical properties and not morphological, with good results despite the resolution limitations [24]. In a different way, a preliminary study on the application of a more complex AI for the identification of SCC on ex vivo confocal scans found a promising performance, with a sensitivity of 76% and a specificity of 91% for distinguishing between SCC and tumor-free regions, endorsing its potential for application in this context [25]. Recently, machine learning AI trained on larger datasets of LC-OCT images have shown remarkable results in scoring cellular atypia [26]. In a recent article, Fischman et al. presented an AI with 135 3D image stacks of LC-OCT. The machine segmented all nuclei and defined cellular spaces. Based on those segmentations, a set of quantifiable, reproducible and interpretable metrics were defined. They then asked clinicians and three different computer-aided approaches (a ruled-based definition, a machine learning unsupervised approach and a weakly supervised approach) to define keratinocyte atypia. When comparing with histological examination as the gold standard, they found that machine learning approaches showed a better discriminative power than the rule-based approach and outperformed medical experts in assessing atypia. The atypia scores provided by the AI matched the histological diagnosis (healthy, subclinical damage, AK and SCC). Figure 2 depicts an example of AI scoring of atypia on LC-OCT images taken from four different AKs. Nuclei are automatically segmented and ranked according to their atypia directly on the 3D image, allowing a view of their spatial distribution. An overall atypia score for the lesion is then provided.

These promising data endorse the potential of AI when applied to non-invasive imaging techniques in the classification of lesions within a cancerization field. Another application to be explored is the prediction of progression from AK to SCC. To our knowledge, no studies on the matter have been published yet. We strongly suggest that these studies should focus on the scoring of the atypia and its localization within the epidermis as these have proven to be histological markers of progression. These advancements may, in the future, help clinicians make choices that will eventually reduce morbidity, mortality and costs.

## 4. AI’s Potential in the Understanding of Cancerization Fields

On another level, genetic alterations are shared between AK and SCC [27]. Cutaneous field cancerization occurs in areas exposed to chronic UVR and leads to many mutations, some unimportant to the process of tumorigenesis. In fact, SCCs rank among tumors with the highest mutational burden, making it difficult to determine which of them are key to their development into cancer within the cancerization field. The tumor suppressor gene TP53, which induces cell cycle arrest and apoptosis, for instance, is the most commonly mutated gene in SCCs but is also found mutated in healthy looking skin [27,28,29]. Likely, the genotype–phenotype is not restricted, and factors other than driver mutations might intervene in cancer development [2]. An example of this could be mutational order. Durinck et al. cleverly studied the temporal order of mutations required for AKs to evolve into invasive SCCs and proposed that TP53 inactivation precedes driver oncogene mutations in AKs that will progress into SCC. Conversely, precursor lesions that activate oncogenes first (before TP53 inactivation) fail to progress [30]. Other clues are the tumor microenvironment, epigenetic alterations and epistatic effects. The risk of progression of a particular AK into SCC cannot then be reduced to its mutational landscape at a particular time point.

Non-invasive imaging technologies have been compared to histological examination at the cellular level. We ask ourselves if AI could expand the application of these technologies to the molecular and genetic levels by identifying morphological or optical patterns related to a particular mutational state. As these techniques evaluate the skin in vivo, lesions could be followed over time and patterns of prognostic significance could potentially be found.

## 5. Conclusions: Future Questions and Pitfalls

The combination of these promising technologies and AI brings us closer to a correct, fast and low-resource-consuming classification of lesions within a cancerization field in a non-invasive manner and in a not-so-distant future, to a progression risk assessment.

In our setting, non-invasive skin imaging has changed our regular practice, reducing the need for biopsy and visits and orienting treatment intervention for otherwise difficult-to-classify lesions. The introduction of AI in this field is exciting and opens up many possibilities as AI can perform tasks impossible for humans in a reasonable amount of time. As highlighted throughout the text, there are questions that remain to be asked, creating opportunities for future studies: (1) Is the degree of keratinocyte atypia related to the risk of progression to SCC? (2) Does the spatial localization of the atypia matter? As previous studies have suggested, basal proliferation and atypia could be used as markers and potentially as future study metrics. (3) Are there metrics other than purely morphological ones, such as optical metrics, that could be used as predictors for the progression of AKs to SCC? In this line, could other phenotypical changes, as suggested by Curtis et al., such as functional ones or those related to the microenvironment, be measured by imaging techniques and detected by AI? (4) Could AI find a correlation between all these metrics and a particular mutational signature?

We do acknowledge some limitations that still need to be overcome to be able to reliably answer these questions. Regarding imaging techniques, depth penetration is still the main worry, particularly for LC-OCT and RCM. The assessment of the whole epidermis as well as the integrity of the dermal–epidermal junction are necessary for the diagnosis of AKs and SCC. If very thick/acanthotic, lesions cannot be correctly assessed in the field of view and a precise diagnosis cannot be made. Engineers working on LC-OCT are addressing this issue and we expect that within two years, technical progress will be made in terms of penetration. Another limitation is the presence of crust or blood. The above-mentioned imaging techniques use the light reflected from the tissue to create images. Crust and blood scatter light, impeding a clear and good-quality acquisition and making image interpretation difficult. From our experience, the application of urea or salicylic acid formulations some weeks before the examination can reduce this inconvenience. Concerning AI, training on large datasets using different patient populations is vital to avoid bias and ensure reproducibility and generalization of its application.

In conclusion, we believe that by harnessing these advancements, clinicians can improve early detection, risk stratification and treatment planning for patients presenting with a cancerization field and a risk of developing SCC. As research continues to expand our understanding of cancerization field’s complex biology, ongoing collaborations between scientists, clinicians and engineers will pave the way for innovative diagnostic and therapeutic approaches, ultimately reducing the morbidity, mortality and costs associated with this disease.
